# Supplementary material for: Genome-wide association meta-analysis of age at onset of walking in over 70,000 infants of European ancestry
Source: Nat Hum Behav. 2025 May 7;9(7):1470–87. doi: 10.1038/s41562-025-02145-1 (PMC12283345; doi:10.1038/s41562-025-02145-1)
Supplement: Supplementary file 2 — Reporting Summary [file 41562_2025_2145_MOESM2_ESM.pdf]

nature portfolio

Corresponding author(s): Angelica Ronald

Last updated by author(s): Dec 20, 2024

Reporting Summary

Nature Portfolio wishes to improve the reproducibility of the work that we publish. This form provides structure for consistency and transparency in reporting. For further information on Nature Portfolio policies, see our Editorial Policies and the Editorial Policy Checklist.

Statistics

For all statistical analyses, confirm that the following items are present in the figure legend, table legend, main text, or Methods section.

n/a	Confirmed	
☐	☒	The exact sample size (*n*) for each experimental group/condition, given as a discrete number and unit of measurement
☐	☒	A statement on whether measurements were taken from distinct samples or whether the same sample was measured repeatedly
☐	☒	The statistical test(s) used AND whether they are one- or two-sided<br>*Only common tests should be described solely by name; describe more complex techniques in the Methods section.*
☐	☒	A description of all covariates tested
☐	☒	A description of any assumptions or corrections, such as tests of normality and adjustment for multiple comparisons
☐	☒	A full description of the statistical parameters including central tendency (e.g. means) or other basic estimates (e.g. regression coefficient) AND variation (e.g. standard deviation) or associated estimates of uncertainty (e.g. confidence intervals)
☐	☒	For null hypothesis testing, the test statistic (e.g. *F*, *t*, *r*) with confidence intervals, effect sizes, degrees of freedom and *P* value noted<br>*Give P values as exact values whenever suitable.*
☐	☒	For Bayesian analysis, information on the choice of priors and Markov chain Monte Carlo settings
☒	☐	For hierarchical and complex designs, identification of the appropriate level for tests and full reporting of outcomes
☐	☒	Estimates of effect sizes (e.g. Cohen's *d*, Pearson's *r*), indicating how they were calculated

Our web collection on statistics for biologists contains articles on many of the points above.

Software and code

Policy information about availability of computer code

Data collection	No software was used for data collection for the purpose of this study.
Data analysis	MoBa phenotype data analysis was performed using the R package phenotools: https://github.com/psychgen/phenotools Genotype data analysis was performed in PLINK 1.9 https://www.cog-genomics.org/plink/ , PLINK 2 https://www.cog-genomics.org/plink/2.0/, GCTA https://yanglab.westlake.edu.cn/software/gcta/#Overview, R. Summary statistics quality control was performed using the R package GWASinspector http://gwasinspector.com/ . The meta-analysis was performed in METAL https://genome.sph.umich.edu/wiki/METAL. Post-GWAS fine mapping and functional annotation was performed in FUMA (version 1.5.2) and MAGMA (version 1.08) https://fuma.ctglab.nl/ . Colocalization analyses were conducted using R libraries locuscomparer 1.0.0 (https://github.com/boxiangliu/locuscomparer), coloc 5.2.2 (https://CRAN.R-project.org/package=coloc) and susieR 0.12.35 (https://github.com/stephenslab/susieR). SNP heritability, genetic correlation and partitioned heritability were estimated using LD score regression https:/ /github.com/bulik/ldsc . Genomic SEM is available here: https:/ /github.com/GenomicSEM/GenomicSEM . The MiXeR program is available here: https:/ /github.com/precimed/mixer. The polygenic scores were calculated using PRS-cs https:/ /github.com/getian107 /PRScs . The scripts for the within and between-family polygenic score analyses are available here: https:/ /github.com/PerlineDemange/GeneticNurtureNonCog/.

For manuscripts utilizing custom algorithms or software that are central to the research but not yet described in published literature, software must be made available to editors and reviewers. We strongly encourage code deposition in a community repository (e.g. GitHub). See the Nature Portfolio guidelines for submitting code & software for further information.

Data

Policy information about availability of data

All manuscripts must include a data availability statement. This statement should provide the following information, where applicable:

- Accession codes, unique identifiers, or web links for publicly available datasets
- A description of any restrictions on data availability
- For clinical datasets or third party data, please ensure that the statement adheres to our policy

The summary statistics of the genome-wide association study of age at onset of walking are available on FigShare (doi: 10.6084/m9.figshare.28071566).

eQTL results for the ROSMAP, Mayo TCX, Mayo CER and cortical meta-analysis from Sieberts et al are available through the AMP-AD Knowledge Portal: https://www.synapse.org/Synapse:syn2580853/wiki/409840

The accession number for the raw RNA-seq and WGS data from BrainVar, along with processed files, is PsychENCODE Knowledge Portal: syn21557948 on Synapse.org (https://www.synapse.org/#!Synapse:syn4921369).

Developing Human Connectome project data is open-access and data are available for download via https://nda.nih.gov/edit_collection.html?id=3955.

Research involving human participants, their data, or biological material

Policy information about studies with human participants or human data. See also policy information about sex, gender (identity/presentation), and sexual orientation and race, ethnicity and racism.

Reporting on sex and gender	Biological sex was used as a co-variate and for sex-stratified analyses in this study. Sex was determined based on sex chromosome information and self or parent-report.
Reporting on race, ethnicity, or other socially relevant groupings	Genetic ancestry was estimated based on principal component analysis on the cohorts' genotype data following standard procedures (e.g., Marees et al., 2018 doi: 10.1002/mpr.1608, Corefield et al., 2022 doi: 10.1101/2022.06.23.496289).
Population characteristics	The participants were 70,560 European-ancestry genotyped children (34,345 males and 33,623 females) from four cohorts: Norwegian Mother, Father and Child Cohort Study (MoBa, N = 58,302), Netherlands Twin Register (NTR, N = 6,251), Lifelines multi-generational prospective population-based birth cohort study (N = 3,415) and Medical Research Council National Study for Health and Development (NSHD, N = 2,592). Genotype data were collected at birth (MoBa), at multiple time points (NTR), during one of the visits for data collection (Lifelines), no information as to when it was collected is provided for the NSHD cohort. Phenotype data were parent-report questionnaires collected when the children were 18 to 36 months for MoBa, after the second birthday of the NTR children (mean age 2.34 years, SD 0.25), during the first assessment of participants, known as the baseline assessment for Lifelines, at age 2 years of the children for NSHD.<br>The Developing Human Connectome Project included newborn infants born in London (UK) across a spread of gestational ages at birth (range: 23 to 43 + 1 weeks + days) and post-menstrual ages at the time of study (range: 26 + 5 to 45 + 1).The subsample included in the current study comprised 264 term-born infants (137 male, 127 female) with available genotype and T2 magnetic resonance images.
Recruitment	For MoBa, participants were recruited through hospitals, firstly in Bergen starting in 1999, and then expanding to 50 of 52 Norway's hospitals with maternity units. NTR is a population-based cohort of over 200,000 people from across the Netherlands. It consists of twin-families, i.e. twins, their parents, spouses and siblings aged between O and 99 years at recruitment. NTR started around 1987 with new-born twins and adolescent and adult twins. The Lifelines study was established in 2006 and asked all the general medical practitioners operating in the provinces of Friesland, Groningen and Drenthe to invite their patients aged between 25 and 50 to take part in the study unless the patient met one of five exclusion criteria (as determined by the practitioner): a) having a severe psychiatric or physical illness (such that the individual was not fully capable to make rational decisions), b) life expectancy of less than 5 years, c) being unable to complete a Dutch language questionnaire, d) a lack of ability in the Dutch language, or e) not being able to visit their medical practitioner (http://wiki-lifelines.web.rug.nl/doku.php?id=cohort). NSHD recruited all women who gave birth in a single week in March 1946 in England, Wales or Scotland. Infants in the Developing Human Connectome Project were recruited at St Thomas' Hospital, London and imaged at the Evelina Newborn Imaging Centre, Centre for the Developing Brain, King's College London, United Kingdom. Pregnant woman with fetal age estimated from last menstrual period and live infants between 23 and 44 weeks gestational age were invited to the study. Mothers or infants with contraindication to Magnetic Resonance imaging, preterm infants who are too unwell to tolerate the scanning period, and language difficulties preventing proper communication about the trial and the consent process.
Ethics oversight	This study and the related secondary data analysis were approved by the Departmental Ethics Committee of the Psychological Science Department of Birkbeck, University of London on 27th October 2020 (reference number 2021007). MoBa and the related data collection was authorised by a licence from the Norwegian Data Protection Agency and an approval from the The Regional Committees for Medical and Health Research Ethics (REK). MoBa is regulated by the Norwegian Health Registry Act. The current study was approved by REK (2016/1702).<br>Informed consent for NTR was obtained from parents or guardians. The study was approved by the Central Ethics Committee on Research Involving Human Subjects of the VU University Medical Centre, Amsterdam, an Institutional Review Board certified by the U.S. Office of Human Research Protections (IRB number IRB00002991 under Federal-wide Assurance-FWA00017598; IRB/institute codes, NTR 03-180).

For Lifelines, participants in Lifelines gave written consent prior to physical examination. The study is conducted according to the principles of the Declaration of Helsinki and in accordance with the UMCG research code and is approved by the medical ethical committee of UMCG (document number METC UMCG METc 2007/152).
For NSHD, the collection of blood samples and DNA information from the participants was approved by ethical approval reference MREC no. 98/2/121.
The Developing Human Connectome Project (dHCP) was approved by the UK Health Research Authority (Research Ethics Committee reference number: 14/LO/1169) and written parental consent was obtained in every case for imaging and open data release of the anonymized data.

Note that full information on the approval of the study protocol must also be provided in the manuscript.

Field-specific reporting

Please select the one below that is the best fit for your research. If you are not sure, read the appropriate sections before making your selection.

☒ Life sciences ☐ Behavioural & social sciences ☐ Ecological, evolutionary & environmental sciences

For a reference copy of the document with all sections, see nature.com/documents/nr-reporting-summary-flat.pdf

Life sciences study design

All studies must disclose on these points even when the disclosure is negative.

Sample size

Cohorts were invited to take part in the GWAS meta-analysis if they had available phenotype data (age at onset of independent walking in months) and genotype for more than 1,000 individuals. The minimum sample size for participanting in the GWAS meta-analysis was defined a priori and pre-registered on OSF (https://osf.io/jyk6d/).
The total sample size for this GWAS meta-analysis was 70,560 infants. With 2,525 SNPs (11 independent loci) passing a genome-wide significance threshold, the current sample demonstrated enough power to detect genetic variation associated with age at onset of walking with p < 5 x 10-8.

Data exclusions

In all the individual cohorts, samples were excluded from the GWAS if they had missing phenotype data, if they presented excess autosomal heterozygosity, mismatch between self-reported and genetic sex, XXV genotype and other aneuploidies, individual genotyping rate< 90%. Duplicate samples and samples whose genetically determined ancestry did not overlay with the European ancestry cluster based on a reference panel were also excluded.

Replication

For colocalization, results were attempted twice: once in cerebellum and once in cortex. Results in RBL2 were replicated in both, and we reported differences in results at genomic locus 6 which has a complex haplotype structure.

Replication of the association between polygenic score (PGS) and infant gross-motor skills was performed on the Developing Human Connectome Project (dHCP) cohort, including 217 European term-born infants that had been assessed using the Bayley-Ill Scales of Infant and Toddler Development at 18-months of age. The relationship between scaled gross motor score and the age at onset of walking PGS was tested using a regression model. Sex, gestational age at birth, birth weight z-score, home environment score (as a proxy for socioeconomic status) and 10 ancestral PCs were included as covariates. We confirmed in this independent sample that the age at onset of walking PGS was significantly associated with lower Bayley's gross motor score, indicating worse/possibly delayed gross motor skills (β = -0.161, SE= 0.070, p = 0.022).

Randomization

There was no randomization involved in this study. This was not a randomized control trial, but an observational design.

Blinding

There was no blinding involved in this study with respect to group allocation, as this was an observational study.

Reporting for specific materials, systems and methods

We require information from authors about some types of materials, experimental systems and methods used in many studies. Here, indicate whether each material, system or method listed is relevant to your study. If you are not sure if a list item applies to your research, read the appropriate section before selecting a response.

Materials & experimental systems

n/a	Involved in the study	
☒	☐	Antibodies
☒	☐	Eukaryotic cell lines
☒	☐	Palaeontology and archaeology
☒	☐	Animals and other organisms
☒	☐	Clinical data
☒	☐	Dual use research of concern
☒	☐	Plants

Methods

n/a	Involved in the study	
☒	☐	ChIP-seq
☒	☐	Flow cytometry
☐	☒	MRI-based neuroimaging

Plants

Seed stocks: N/A

Novel plant genotypes: N/A

Authentication: N/A

Magnetic resonance imaging

Experimental design

Design type: N/A

Design specifications: N/A

Behavioral performance measures: N/A

Acquisition

Imaging type(s): structural

Field strength: 3T

Sequence & imaging parameters: T2-weighted images were obtained using a Turbo Spin Echo sequence, acquired in two stacks of 2D slices (in sagittal and axial planes), using parameters: TR=12s, TE=156ms, SENSE factor 2.11 (axial) and 2.58 (sagittal) with overlapping slices (resolution 0.8x0.8xl.6mm3).

Area of acquisition: whole brain scan

Diffusion MRI: ☐ Used ☒ Not used

Preprocessing

Preprocessing software: Advanced Neuroimaging Tools (ANTs)

Normalization: The log-Jacobian determinant images were calculated by applying ANTs algorithms to the non-linear transformation deformation tensor fields.

Normalization template: 40-week dHCP neonatal atlas (https://brain-development.org/brain-atlases/atlases-from-the-dhcp-project/)

Noise and artifact removal: *Describe your procedure(s) for artifact and structured noise removal, specifying motion parameters, tissue signals and physiological signals (heart rate, respiration).*

Volume censoring: *Define your software and/or method and criteria for volume censoring, and state the extent of such censoring.*

Statistical modeling & inference

Model type and settings: Permutation testing using the randomise function, part of the FMRIB Software Library (FSL) was used with a general linear model.

Effect(s) tested: beta estimate of a linear regression.

Specify type of analysis: ☐ Whole brain ☒ ROI-based ☐ Both

Anatomical location(s): *Describe how anatomical locations were determined (e.g. specify whether automated labeling algorithms or probabilistic atlases were used).*

Statistic type for inference: voxel-wise

(See Eklund et al. 2016)

Correction	Threshold-Free Cluster Enhancement (TFCE) and Family-Wise Error (FWE) rate were applied to correct for multiple comparisons between voxels.

Models & analysis

n/a	Involved in the study
☒	☐ Functional and/or effective connectivity
☒	☐ Graph analysis
☐	☒ Multivariate modeling or predictive analysis

Multivariate modeling and predictive analysis	Independent variable: polygenic score. Covariates: gestational age, postmenstrual age at scan, sex, weight-z-score and 10 ancestral principal components as covariates
